# REACT-UTI: A 72-Hour Composite to Predict Early Non-Response and Length of Stay in Hospitalized Adults with Lower Urinary Tract Infection—A Prospective Observational Study

**DOI:** 10.3390/biomedicines13122870

**Published:** 2025-11-25

**Authors:** Adela Benea, Lavinia Stelea, Mirela Turaiche, Iulia Bogdan, Livia Stanga, Daniel-Florin Lighezan, Ciprian Rachieru, Felicia Marc, Oana Silvana Sarau, Cristian Andrei Sarau

**Affiliations:** 1 Doctoral School, Faculty of Medicine, “Victor Babes” University of Medicine and Pharmacy Timisoara, 300041 Timisoara, Romania; adela.benea@umft.ro; 2 Methodological and Infectious Diseases Research Center, Faculty of Medicine, “Victor Babes” University of Medicine and Pharmacy Timisoara, 300041 Timisoara, Romania; paliu.mirela@umft.ro (M.T.); iulia-georgiana.bogdan@umft.ro (I.B.); 3 Department of Obstetrics and Gynecology, Faculty of Medicine, “Victor Babes” University of Medicine and Pharmacy Timisoara, 300041 Timisoara, Romania; stelea.lavinia@umft.ro; 4 Discipline of Microbiology, Faculty of Medicine, “Victor Babes” University of Medicine and Pharmacy Timisoara, 300041 Timisoara, Romania; 5 Department I of Internal Medicine, Faculty of Medicine, “Victor Babes” University of Medicine and Pharmacy Timisoara, 300041 Timisoara, Romania; dlighezan@umft.ro (D.-F.L.); ciprian.rachieru@umft.ro (C.R.); csarau@umft.ro (C.A.S.); 6 Center for Advanced Research in Cardiovascular Pathology and Hemostaseology, “Victor Babes” University of Medicine and Pharmacy Timisoara, 300041 Timisoara, Romania; 7 Department of Medical Sciences, Faculty of Medicine and Pharmacy, University of Oradea, 410073 Oradea, Romania; 8 Department V of Internal Medicine, Discipline of Hematology, “Victor Babes” University of Medicine and Pharmacy Timisoara, 300041 Timisoara, Romania; oana.sarau@umft.ro

**Keywords:** urinary tract infections, catheter-related infections, C-reactive protein, antimicrobial resistance, anti-bacterial agents/therapeutic use

## Abstract

**Background and Objectives:** Early bedside tools that flag non-response in hospitalized adults with lower urinary tract infection (UTI) could align clinical care with antimicrobial stewardship. We evaluated REACT-UTI, a 72 h composite combining C-reactive protein (CRP) clearance ≥35%, defervescence (temperature < 37.5 °C), and ≥2-point symptom improvement, to predict early non-response and hospital length of stay (LOS), and we assessed modifiable processes of care. **Methods:** We conducted a prospective observational study of adults with culture-confirmed lower UTI (n = 126) admitted to a tertiary hospital in Timișoara (December 2023–August 2025). The primary outcome was 72 h early clinical response (ECR) defined by REACT-UTI. Multivariable logistic regression examined associations of catheter-associated UTI (CAUTI), time-to-effective therapy, baseline CRP, diabetes, early catheter removal/exchange (≤48 h), and early intravenous-to-oral switch (≤72 h) with non-response. **Results:** Overall, 76/126 patients (60.3%) achieved ECR. Non-responders more often had CAUTI, higher baseline CRP, longer time-to-effective therapy, ESBL or fluoroquinolone-resistant *Enterobacterales*, and longer LOS (14.1 vs. 9.8 days; *p* < 0.001). Adjusted models showed that CAUTI, delayed active therapy, higher baseline CRP, and diabetes increased the odds of non-response, whereas early catheter removal (adjusted odds ratio [aOR] 0.5, 95% confidence interval [CI] 0.3–0.9) and early IV-to-oral switch (aOR 0.4, 0.2–0.8) were protective. Greater CRP clearance correlated with shorter LOS (ρ = −0.52; *p* < 0.001). **Conclusions:** In this single-center setting with a high burden of antimicrobial resistance, REACT-UTI at 72 h identified patients at risk of early non-response and prolonged hospitalization and highlighted actionable levers—timely active therapy, catheter management, and early oral step-down. External validation in diverse settings is needed before broader implementation.

## 1. Introduction

Hospitalized adults with lower urinary tract infection (UTI) represent a clinically diverse population in whom outcomes vary with host factors, device exposure, and local resistance patterns. Catheter-associated UTI (CAUTI) contributes substantially to hospital-acquired infection (HAI) burden and prolonged recovery, while community-onset cases are increasingly complicated by prior antibiotic exposure and comorbidities [1,2]. Pragmatic, early indicators that flag patients unlikely to respond to initial therapy are needed to direct stewardship and source-control decisions in real time.

Across Europe, antimicrobial resistance (AMR) among *Enterobacterales* remains high, with *E. coli* and *Klebsiella pneumoniae* driving the majority of bloodstream infections involving resistance; more than half of invasive *E. coli* isolates in 2023 were resistant to ≥1 key antimicrobial group, and combined resistance remains frequent [3]. Beyond bloodstream infections, recent reviews highlight high and rising resistance rates in uropathogenic *K. pneumoniae*, including the contribution of siderophore-mediated virulence and resistance mechanisms. Likewise, contemporary syntheses of antimicrobial resistance in *E. coli* underscore widespread resistance to key oral agents and the increasing prevalence of extended-spectrum β-lactamase (ESBL)-producing strains, particularly in urinary isolates [3,4,5]. Globally, *E. coli* and *K. pneumoniae* remain the predominant causes of both community- and hospital-acquired UTI, as illustrated by laboratory-based surveillance from Lebanon and other regions reporting these species as the most frequent uropathogens and key drivers of resistance profiles [6,7]. Similar resistance patterns have been reported in surveillance studies from the Middle East, Africa, and Asia, where urinary *E. coli* and *Klebsiella* spp. frequently exhibit high rates of resistance to fluoroquinolones and third-generation cephalosporins, underscoring the global relevance of timely active therapy and structured early response assessment [6,7,8,9,10].

In Western Romania, recent cohort work delineated differences between CAUTI and non-catheter UTIs and highlighted the interplay among pathogen profiles, inflammatory response, and hospital trajectory. That analysis showed longer stays and more resistant flora in catheterized patients, motivating a shift from static admission variables to dynamic, early response assessment during hospitalization [4].

Contemporary stewardship frameworks explicitly promote a 48–72 h “antibiotic time-out” to reassess diagnosis, cultures, route, and opportunities to de-escalate or discontinue therapy [5]. Embedding such a checkpoint within a disease-specific early-response tool for UTI could harmonize bedside decision-making with stewardship goals and reduce unnecessary intravenous (IV) days [5].

Biomarker trajectories—particularly C-reactive protein (CRP) decline—capture treatment–pathogen–host interactions more completely than single time-point values. In acute pyelonephritis, failure to defervesce by 72 h and persistent systemic signs have been associated with early clinical failure [6], while steeper early CRP decreases correlate with favorable response [7,8,9]. Such kinetics, together with symptom improvement, can be operationalized into an early-response composite suitable for routine wards [6,7,8,9]. Beyond CRP, several other early markers have been proposed to evaluate response in complicated UTI and CAUTI, including time to defervescence, resolution of lower urinary symptoms, and organ dysfunction measures, such as the Sequential Organ Failure Assessment (SOFA) score in septic presentations. Procalcitonin and other inflammatory biomarkers have also been explored as adjuncts to guide the duration of therapy. However, many of these indices require intensive monitoring, are not routinely available on general wards, or lack standardized thresholds in lower UTI [6,7,8,9].

Time-to-effective therapy is a second, modifiable driver of outcome. Delays in active treatment for infections caused by resistant Gram-negatives—particularly carbapenemase-producing strains—have been linked to increased mortality and prolonged hospitalization, reinforcing the need for rapid reassessment when initial empiric coverage is inactive [6,10].

Concurrently, source control for CAUTI—prompt catheter removal or exchange—is fundamental but inconsistently executed within the first 48 h. Long-standing guidelines and implementation studies show that early removal via nurse-driven protocols reduces catheter days and lowers CAUTI rates, and large multicenter prevention programs emphasize catheter stewardship as a cornerstone of quality care [1,2,11,12].

Finally, early IV-to-oral switch after clinical stabilization is safe for many patients with Gram-negative bacteremia of urinary origin and is associated with shorter length of stay—supporting a standardized 72 h checkpoint that includes route optimization when an active oral option exists [13,14].

Therefore, the current study was designed to evaluate a 72 h composite—REACT-UTI (Response Evaluation After Catheter/antibiotic Timing for UTI)—that merges CRP clearance, defervescence, and symptom improvement. The objectives were to (1) validate REACT-UTI against 72 h non-response and LOS; (2) quantify independent effects of CAUTI, time-to-effective therapy, early catheter removal (≤48 h), and IV-to-oral switch (≤72 h) on early response; and (3) describe pathogen and resistance patterns in relation to early response. REACT-UTI is intended to provide a pragmatic, disease-specific 72 h checkpoint that couples a simple physiologic–inflammatory composite with clearly actionable stewardship levers in routine ward care, extending concepts from acute pyelonephritis and sepsis to hospitalized lower UTI.

## 2. Materials and Methods

### 2.1. Study Design and Setting

This prospective observational study enrolled consecutive adults admitted with lower UTI to Victor Babeș Hospital for Infectious Diseases, Timișoara, a tertiary referral center serving Western Romania. Enrollment spanned from December 2023 to August 2025. Clinical care followed hospital protocols; no interventions were mandated by the study. Participants were followed prospectively from hospital admission through the end of the index hospitalization (discharge or in-hospital death); no post-discharge follow-up was performed.

The ethics committee approved the protocol and waived individual consent, given the minimal risk and use of routinely collected data. Patient confidentiality was preserved through de-identification and secure data handling, consistent with national regulations and the Declaration of Helsinki.

### 2.2. PICO Statement

In a prospective, single-center cohort of adults (≥18 years) hospitalized with culture-confirmed lower urinary tract infection at Victor Babeș Hospital, Timișoara (n = 126), the exposures (I) of interest were modifiable, early care processes: time-to-effective antimicrobial therapy (measured in hours from admission), early catheter removal/exchange among catheterized patients (≤48 h), and early IV-to-oral antibiotic switch (≤72 h), with catheter-associated UTI (CAUTI) status treated as a contextual exposure. These were compared (C) with usual care counterparts—longer time-to-effective therapy, no early catheter removal, no early IV-to-oral switch, and non-catheter UTI—to estimate their association with early recovery. The primary outcome (O) was 72 h early clinical response defined by the REACT-UTI composite (CRP clearance ≥35%, temperature < 37.5 °C, and ≥2-point improvement on a 0–10 symptom scale), while secondary outcomes included hospital length of stay (days) and ICU transfer during the index admission; organism profiles and resistance (e.g., ESBL, fluoroquinolone resistance) were evaluated as explanatory endpoints. The time horizon (T) for the primary assessment was 72 h after treatment initiation, with secondary outcomes measured through discharge.

### 2.3. Population, Definitions, and Microbiology

Inclusion criteria were age ≥ 18 years; dysuria/urgency/frequency/suprapubic pain with no flank pain; positive urine culture (≥10^5^ CFU/mL, or ≥10^4^ CFU/mL in symptomatic patients) with ≤2 uropathogens; and antibiotic therapy initiated during admission. Exclusion criteria were pregnancy/postpartum ≤6 weeks; polymicrobial cultures >2 organisms; upper UTI/pyelonephritis; and concurrent non-urinary infection driving systemic signs.

CAUTI was defined as an indwelling urethral catheter in place for ≥48 h prior to symptom onset. Time-to-effective therapy (hours) measured from admission to the first dose of an antibiotic with in vitro activity against the index isolate. Early catheter removal denoted removal or exchange ≤48 h after admission among catheterized patients. Early IV-to-oral switch was any transition to a bioavailable oral agent ≤72 h, guided by hemodynamic stability and oral tolerance.

Urine specimens were collected aseptically and processed according to internal standard operating procedures. Quantitative cultures were performed on routine urinary media with incubation at 35–37 °C for 18–24 h, and colony counts were interpreted using conventional thresholds (≥10^5^ colony-forming units [CFUs]/mL, or ≥10^4^ CFUs/mL in symptomatic patients). Species identification relied on conventional biochemical tests and/or an automated identification system, and antimicrobial susceptibility testing was performed by disk diffusion and/or broth microdilution, interpreted according to contemporary Clinical and Laboratory Standards Institute (CLSI) breakpoints. The ESBL phenotype among *Enterobacterales* was assessed using the CLSI-recommended screening and confirmatory combination-disk approach, and suspected carbapenemase producers underwent additional phenotypic testing in line with national algorithms.

Because several individual uropathogen species were infrequent, resistance analyses were prespecified at the *Enterobacterales* level (ESBL, fluoroquinolone resistance) rather than by individual species to preserve statistical power.

### 2.4. Outcomes and the 72 h REACT-UTI Composite

The primary outcome was 72 h early clinical response (ECR) versus non-response (ECR−). REACT-UTI is an a priori composite developed by the authors, informed by prior data on CRP kinetics and 72 h clinical response in acute pyelonephritis and sepsis cohorts [6,7,8,9,15,16]. ECR (responders) required all three at 72 h: (i) CRP clearance ≥35% from baseline, (ii) temperature < 37.5 °C, and (iii) ≥2-point improvement on a 0–10 UTI symptom scale (dysuria/urgency composite). This study represents the first prospective validation of REACT-UTI in hospitalized adults with lower UTI.

The CRP clearance threshold of ≥35% over 72 h was chosen a priori based on prior reports suggesting that approximately 30–40% of early CRP reductions distinguish favorable from unfavorable trajectories in acute urinary and other bacterial infections [6,7,8].

### 2.5. Sample Size Considerations

This was a pragmatic, single-center, prospective observational study. No formal a priori sample size calculation was performed; instead, we planned to enroll all consecutive eligible patients with lower UTI over the 21-month study period to ensure adequate precision around early response estimates. The resulting sample of 126 patients provided sufficient events for multivariable modeling with the prespecified covariates, as reflected by reasonably narrow confidence intervals around the main adjusted odds ratios. We acknowledge that the study was not powered to detect small differences in less frequent outcomes or rare resistance phenotypes.

### 2.6. Statistical Analysis

Normality was evaluated with the Shapiro–Wilk test. Continuous variables were summarized as mean ± SD (or median [IQR]) and compared by Welch’s *t*-test or the Mann–Whitney U test, as appropriate. Categorical variables used χ^2^ or Fisher’s exact tests. Correlations used Spearman’s ρ. A multivariable logistic regression modeled the odds of 72 h non-response, including clinically relevant covariates (CAUTI; time-to-effective therapy per 6 h; baseline CRP per 20 mg/L; diabetes; early catheter removal; early IV-to-oral switch). Model calibration used the Hosmer–Lemeshow test; performance used Nagelkerke R^2^. Two-sided α = 0.05. Analyses were performed in R 4.3.1.

## 3. Results

Overall, 126 adults with culture-confirmed lower UTI were enrolled. The mean age was 61.8 ± 14.1 years, and 67/126 (53.2%) were male. Diabetes was present in 36/126 (28.6%), and chronic kidney disease was present in 20/126 (15.9%). Just over half of infections were hospital-acquired (66/126, 52.4%), and 57/126 (45.2%) met the criteria for CAUTI. Baseline CRP averaged 83.9 ± 32.4 mg/L, and the mean length of stay for the cohort was 11.6 ± 3.7 days (Table 1).

In Table 2, catheter status delineated expected microbiologic patterns. *E. coli* was less common with catheters (28.1%) than without (47.8%; *p* = 0.024), reflecting biofilm ecology and prior antibiotic exposure that select for non-fermenters and more resistant *Enterobacterales* among CAUTI. Although *P. aeruginosa* showed a numerically higher frequency in CAUTI (14.0% vs. 5.8%), this difference did not reach significance (*p* = 0.117), likely due to sample size. *K. pneumoniae* and *Enterococcus* spp. proportions were comparable across strata. Mixed/other isolates clustered slightly in CAUTI (19.3% vs. 14.5%, *p* = 0.472), consistent with device-related polymicrobial colonization. Among *Enterococcus* isolates, no vancomycin-resistant enterococci (VRE) were detected.

Table 3 links early non-response to resistance phenotype among *Enterobacterales*. ESBL positivity was nearly twice as common in ECR− (44.7%) as ECR+ (22.2%; *p* = 0.022), implying that initial empirical therapy in ECR− was more likely inactive or suboptimal before the susceptibility results arrived. Fluoroquinolone resistance followed the same pattern (47.4% vs. 25.9%; *p* = 0.033). Carbapenem resistance was uncommon in both groups (≤10.5%; *p* = 0.376).

Table 4 operationalizes REACT-UTI’s core premise: what changes by 72 h matters most. Responders achieved robust CRP clearance (46.3%) versus minimal decline in non-responders (12.7%; *p* < 0.001), normalized temperature (36.9 °C vs. 37.7 °C; *p* < 0.001), and greater symptom improvement (+3.6 vs. +1.1 points; *p* < 0.001). These physiologic gains aligned with earlier active therapy (10.7 vs. 22.9 h; *p* < 0.001), confirming that timeliness of correct coverage drives the early biological signal. Process metrics distinguished groups: early IV-to-oral step-down (≤72 h) occurred in 55.3% of ECR+ vs. 12.0% of ECR− (*p* < 0.001), while early catheter removal among catheterized patients was 68.0% vs. 31.0% (*p* = 0.005).

Table 5 confirms that both exposure and timing independently shape the 72 h trajectory. After adjustment, CAUTI nearly doubled the odds of non-response (aOR 1.9), while each 6 h delay in effective therapy raised the odds by 50% (aOR 1.5). Higher baseline CRP modestly increased risk (aOR 1.3 per 20 mg/L), consistent with larger inflammatory burdens requiring more time to resolve. Diabetes retained significance (aOR 1.8), plausibly via impaired neutrophil function and vascular compromise. Crucially, early catheter removal was protective (aOR 0.5), as was early IV-to-oral switch (aOR 0.4).

**Figure 1:**
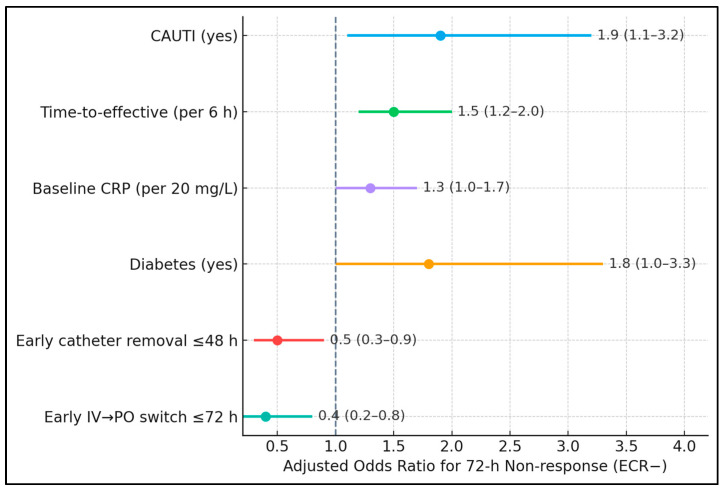
Forest plot with adjusted predictors of 72 h non-response.

Non-response odds were higher with CAUTI (aOR 1.9, 95% CI 1.1–3.2), longer time-to-effective therapy per 6 h (aOR 1.5, 1.2–2.0), baseline CRP per 20 mg/L (aOR 1.3, 1.0–1.7), and diabetes (aOR 1.8, 1.0–3.3). Protective associations included early catheter removal ≤48 h (aOR 0.5, 0.3–0.9) and early IV → PO switch ≤72 h (aOR 0.4, 0.2–0.8). Visually, all risk factors plotted to the right of the null (OR = 1), while protective ones lay to the left, with non-overlapping confidence intervals for the most influential variables (time-to-effective therapy and early IV → PO), as presented in Figure 1.

Table 6 quantifies relationships underpinning REACT-UTI. Greater CRP clearance strongly correlated with shorter LOS (ρ = −0.52; *p* < 0.001), reinforcing the construct’s biological validity: faster dampening of systemic inflammation aligns with accelerated clinical recovery and discharge readiness. Conversely, longer time-to-effective therapy correlated with longer LOS (ρ = +0.41; *p* < 0.001), capturing the downstream impact of initial regimen mismatches when resistant organisms are involved. Baseline CRP related modestly to LOS (ρ = +0.28; *p* = 0.002), consistent with Table 5, while age had a weaker association (ρ = +0.18; *p* = 0.048), suggesting that what happens early in treatment (coverage timing and response) outweighs fixed demographics. Importantly, time-to-effective therapy correlated inversely with CRP clearance (ρ = −0.36; *p* < 0.001).

**Figure 2:**
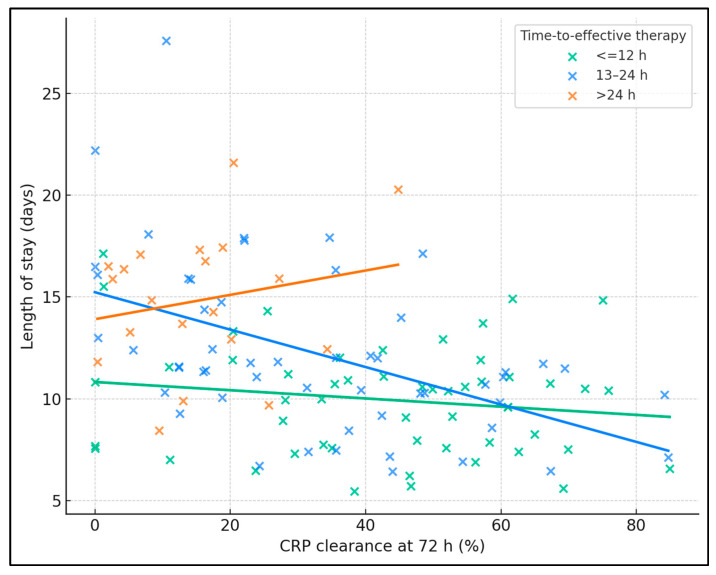
CRP clearance vs. length of stay, stratified by time-to-effective therapy.

Across the cohort, higher 72 h CRP clearance correlated with shorter LOS (Spearman’s ρ −0.44, *p* < 0.001). The 13–24 h stratum showed a clear inverse relationship (ρ −0.53, *p* < 0.001), with regression slope indicating progressively shorter stays as clearance rose, while the ≤12 h group showed a flatter but still negative trend (ρ −0.11, *p* = 0.44), consistent with already low LOS when therapy was timely. In contrast, the >24 h group displayed a weak, non-significant pattern (ρ 0.14, *p* = 0.55) and higher LOS values overall, illustrating that delays blunt the benefit of biomarker improvement (Figure 2).

Table 7 examines how delays to active therapy are mapped onto early response and length of stay. Patients who received an active agent within 12 h had an ECR+ rate of 84.6% and a mean LOS of 10.2 days, whereas those who started after 24 h had an ECR+ rate of 25.0% and stayed 13.8 days on average. The graded pattern was statistically robust: ECR proportions differed markedly across strata (χ^2^ *p* < 0.001), and LOS increased stepwise (Kruskal–Wallis *p* = 0.007). Pairwise testing confirmed that ≤12 h and >24 h strata diverged significantly in LOS (*p* = 0.0069), while intermediate delays (13–24 h) showed numerically longer stays versus ≤12 h but without multiplicity-adjusted significance.

Within the catheterized subset, early device management was strongly associated with better 72 h outcomes. Two-thirds (67.9%) of patients who had removal/exchange within 48 h achieved ECR+, compared with 31.0% without early source control. The effect was statistically significant (Fisher *p* = 0.008) and clinically meaningful (RR 2.19; 95% CI 1.20–3.98), indicating that timely catheter action nearly doubled the chance of early response, as presented in Table 8.

**Figure 3:**
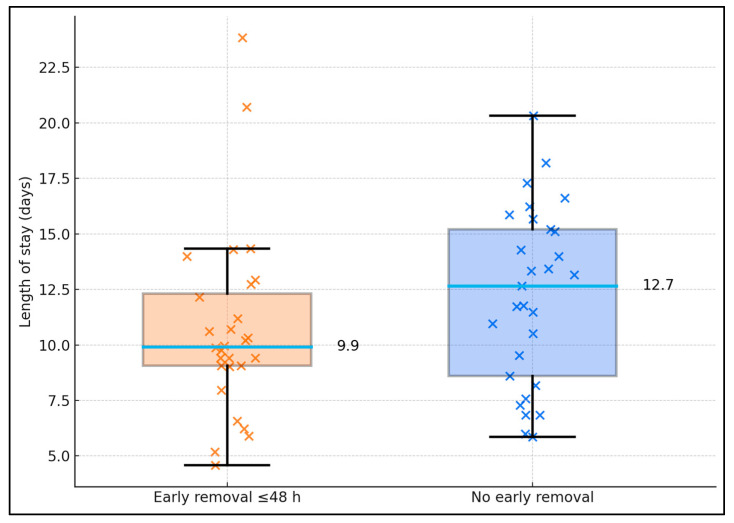
CAUTI subset: length of stay by early catheter removal (≤48 h) vs. no early removal.

Among CAUTI (n = 57), early catheter removal was associated with shorter stays. LOS averaged 9.9 ± 3.8 days with early removal (n = 28) vs. 12.7 ± 3.1 days without (n = 29); Mann–Whitney *p* = 0.0038. Distribution spread demonstrating fewer long-stay outliers when the catheter was removed/exchanged by 48 h—aligning with the CAUTI aOR 0.5 (non-response) in the adjusted model (Figure 3).

## 4. Discussion

### 4.1. Literature Findings

The present findings supported the clinical utility of a standardized 72 h checkpoint for hospitalized lower UTI. Patients who failed the REACT-UTI composite (CRP clearance ≥35%, defervescence, and symptom improvement) had substantially higher inflammatory burden, delays to active therapy, and longer LOS. The 60% 72 h response rate and approximately 4-day LOS difference we observed between responders and non-responders are broadly consistent, with 72 h failure rates and LOS gaps reported in multicenter cohorts of acute pyelonephritis and complicated UTI, in which 20–40% of patients fail to defervesce by 72 h and experience longer hospitalizations [6,7,15,16].

At a population level, antimicrobial resistance imposes substantial excess morbidity, mortality, and healthcare costs, particularly for Gram-negative infections in which therapeutic options are increasingly constrained. In parallel with stewardship and source-control strategies, novel agents are being developed that exploit bacterial nutrient uptake systems, including antibiotics targeting bacterial metallophores (siderophore-like molecules) to enhance drug delivery into Gram-negative pathogens. Such “metallophore-linked” agents illustrate how leveraging iron acquisition pathways can partially overcome conventional resistance mechanisms and may complement efforts to optimize empiric therapy and minimize delays to effective treatment [16]. Related to metallophore-linked agents, Trojan horse antibiotics couple an antimicrobial to a siderophore or peptide vector that is actively transported into bacterial cells, thereby increasing intracellular exposure and potentially lowering the minimum inhibitory concentration required for activity. Early clinical experience with such conjugates, including siderophore–β-lactam combinations, suggests that this strategy can restore susceptibility in some multidrug-resistant Gram-negative infections [16]. While our study focuses on optimizing currently available agents through timely active therapy, catheter stewardship, and IV-to-oral switch, the emergence of Trojan horse antibiotics underscores the need to integrate novel drugs into stewardship frameworks that prioritize rapid, appropriate coverage.

Beyond a single vital sign or value, early-response composites similar to REACT-UTI (incorporating temperature and clinical stability) have performed well in other infection cohorts, supporting the construct validity of our multidomain approach [17]. Furthermore, the strong inverse association between CRP clearance and LOS paralleled randomized data showing that CRP-guided management in Gram-negative bacteremia can safely shorten antibiotic exposure without compromising outcomes, reinforcing the biological relevance of early inflammatory trajectories [18].

Observed microbiology differences by catheter status also mirrored the contemporary literature. As in our cohort, catheterized patients exhibited a lower share of *E. coli* and a broader distribution of non-*E. coli* uropathogens, consistent with biofilm ecology and prior antibiotic exposure [19]. Large molecular surveys of catheter biofilms further depict polymicrobial communities with *Enterobacterales*, *Pseudomonas*, and *enterococci*, explaining our numerically higher—but not statistically significant—Pseudomonas signal in CAUTI [20]. These ecological patterns rationalize why catheter stewardship (timely removal or exchange) was associated with better early response in our data and underscore the importance of device management as part of the 72 h review.

Resistance phenotypes tracked closely with early non-response. ESBL and fluoroquinolone resistance were markedly more frequent among ECR− patients, consistent with population-based observations of rising ESBL prevalence in *E. coli* urinary and bloodstream infections and with the clinical burden these mechanisms impose (treatment mismatches and longer LOS) [21]. Our adjusted analysis identified time-to-effective therapy as the strongest modifiable predictor of non-response; this dovetailed with multicenter cohorts and systematic reviews demonstrating that delays to appropriate coverage worsen outcomes in Gram-negative infections, largely by prolonging uncontrolled bacterial replication and systemic inflammation [22,23]. Together, these data argue for integrating rapid susceptibility reporting and early active-agent optimization into the 72 h bundle when initial empiric therapy is inactive. We did not construct a composite multidrug-resistant (MDR) classification; instead, we focused on individual resistance phenotypes that are readily interpretable at the bedside (ESBL and fluoroquinolone resistance). As a result, we may underestimate the burden of MDR *Enterobacterales*, and future work should apply standardized MDR definitions to better quantify this burden. Species-specific resistance comparisons, particularly for less common uropathogens, were considered exploratory and were not pursued in adjusted analyses because of limited sample sizes and the risk of overfitting.

Diabetes remained independently associated with early non-response after covariate adjustment, which aligned with mechanistic and epidemiologic data. Type 2 diabetes increases susceptibility to more severe and recurrent UTIs through impaired neutrophil function, autonomic bladder dysfunction, and a propensity for infections due to resistant organisms [24]. Recent syntheses also suggest higher recurrence risks among diabetics, reinforcing that host-factor context (glycemic control and complications) modifies the expected speed of recovery and may justify a lower threshold for imaging or for extending observation before declaring success [25]. Within REACT-UTI, this supports a nuanced interpretation of the 72 h composite in metabolic disease (confirming adequate source control and oral absorption before step-down).

Process-of-care variables central to CAUTI prevention were strongly associated with benefit in our cohort. Early catheter removal or exchange (≤48 h) nearly doubled the probability of meeting the 72 h response composite and shortened LOS, in line with prior evidence that reminder/stop-order and nurse-driven removal protocols reduce catheter days and CAUTI events and improve patient safety [26,27,28]. These implementation strategies are low-cost and scalable, and their measurable impact on early response in our study suggests that embedding a hard stop for catheter reassessment into the 72 h review could accelerate recovery and reduce downstream complications.

Finally, early IV-to-oral switch (≤72 h) was independently protective, echoing large multicenter data showing that most patients with Gram-negative bacteremia—predominantly of urinary origin—achieve clinical stability by day 5 and can transition safely to oral agents when an active option exists [29]. Contemporary cohort and meta-analytic work further indicate that oral step-down (including β-lactams when dosed appropriately) achieves outcomes comparable to continued IV therapy and often shortens LOS [30,31]. Complementary trials and meta-analyses support shorter total durations (≈7 days) for uncomplicated urinary-source bacteremia, provided source control and early clinical improvement are present, which aligns with our stewardship-oriented 72 h composite [32].

Taken together, these findings suggest that a simple, 72 h composite grounded in routine clinical data can meaningfully stratify early non-response and length of stay in hospitalized lower UTI while highlighting specific, modifiable processes of care. Rather than functioning solely as a prognostic score, REACT-UTI can be viewed as a bedside scaffold to structure the 48–72-h “antibiotic time-out” around three actionable domains: timely active therapy, catheter/source control, and IV-to-oral step-down. Future multicenter validation in diverse resistance ecologies, coupled with implementation and health–economic studies, will be essential to determine whether embedding REACT-UTI into stewardship pathways improves patient outcomes and optimizes resource use.

### 4.2. Strengths and Limitations

Several limitations warrant consideration. First, although data collection was prospective, the single-center design in a tertiary hospital with a relatively high prevalence of resistant *Enterobacterales* may limit generalizability to settings with different case mix, device utilization, or resistance ecology. Second, REACT-UTI relies on serial CRP, which, while widely available in many European centers, may be less accessible elsewhere and can be influenced by non-infectious inflammatory processes; the requirement for concordant defervescence and symptom improvement partially mitigates misclassification. Third, time-to-effective therapy was derived from electronic medication administration and laboratory timestamps; despite standardized workflows, some residual timing imprecision is possible. Fourth, we focused on lower UTI and excluded pyelonephritis, so the composite should not be extrapolated to upper tract or bacteremic infections without dedicated validation. Fifth, empiric regimens were not protocolized, and we lacked a validated baseline severity score beyond CRP and vital signs; residual confounding of the association between time-to-therapy, CRP, and outcomes, therefore, cannot be excluded. Finally, the study was not powered to detect small differences in uncommon outcomes or to fully characterize MDR patterns, and species-specific resistance analyses were limited by sample size.

## 5. Conclusions

In this prospective observational study, a standardized 72 h composite—REACT-UTI, integrating CRP clearance ≥35%, defervescence (<37.5 °C), and ≥2-point symptom improvement—reliably identified early non-response and stratified hospital length of stay. Non-response was independently associated with catheter-associated infection, longer time-to-effective therapy, higher baseline inflammatory burden, and diabetes, whereas timely catheter removal (≤48 h) and early IV-to-oral switch (≤72 h) were protective. Model calibration and explanatory power supported clinical utility, and the inverse correlation between CRP clearance and length of stay reinforced biological plausibility. Embedding REACT-UTI within the 48–72 h “antibiotic time-out” offers a pragmatic scaffold to prompt coverage optimization, device stewardship, and route de-escalation. External, multicenter validation across diverse resistance ecologies, together with implementation and health–economic studies, will be essential before the broader adoption of REACT-UTI into routine stewardship pathways.

## Figures and Tables

**Table 1:** Baseline characteristics by 72 h early clinical response (ECR).

Characteristic	ECR+ (n = 76)	ECR− (n = 50)	*p*-Value
Age, years (mean ± SD)	60.4 ± 14.2	63.9 ± 13.8	0.171
Male sex, n (%)	39 (51.3)	28 (56.0)	0.606
Diabetes, n (%)	16 (21.1)	20 (40.0)	0.021
Chronic kidney disease, n (%)	11 (14.5)	9 (18.0)	0.596
Hospital-acquired UTI, n (%)	34 (44.7)	32 (64.0)	0.034
Catheter-associated UTI (CAUTI), n (%)	28 (36.8)	29 (58.0)	0.02
Baseline CRP, mg/L (mean ± SD)	74.3 ± 28.7	98.5 ± 32.4	<0.001
Length of stay, days (mean ± SD)	9.8 ± 2.7	14.1 ± 3.5	<0.001

Abbreviations: CRP, C-reactive protein; UTI, urinary tract infection; ECR, early clinical response.

**Table 2:** Pathogen distribution by catheter status.

Pathogen	CAUTI (n = 57), n (%)	Non-Catheter (n = 69), n (%)	*p*-Value *
*Escherichia coli*	16 (28.1)	33 (47.8)	0.024
*Klebsiella pneumoniae*	11 (19.3)	10 (14.5)	0.47
*Pseudomonas aeruginosa*	8 (14.0)	4 (5.8)	0.117
*Enterococcus* spp.	5 (8.8)	4 (5.8)	0.535
*Proteus* spp.	6 (10.5)	8 (11.6)	0.855
Mixed/other	11 (19.3)	10 (14.5)	0.472

* *p*-values compare proportion within pathogen (CAUTI vs. non-catheter). Abbreviations: CAUTI, catheter-associated urinary tract infection.

**Table 3:** *Enterobacterales* resistance by early response status.

Resistance Phenotype (*Enterobacterales* Only)	ECR+ (n = 54), n (%)	ECR− (n = 38), n (%)	*p*-Value
ESBL screen positive	12 (22.2)	17 (44.7)	0.022
Fluoroquinolone resistance	14 (25.9)	18 (47.4)	0.033
Carbapenem resistance	3 (5.6)	4 (10.5)	0.376
Aminoglycoside resistance	10 (18.5)	12 (31.6)	0.148
Trimethoprim–sulfamethoxazole resistance	16 (29.6)	17 (44.7)	0.137

Abbreviations: ECR, early clinical response; ESBL, extended-spectrum β-lactamase.

**Table 4:** Dynamic 72 h changes and care processes by early response status.

Variable	ECR+ (n = 76)	ECR− (n = 50)	*p*-Value
CRP clearance, % (mean ± SD)	46.3 ± 18.1	12.7 ± 14.9	<0.001
Temperature at 72 h, °C (mean ± SD)	36.9 ± 0.4	37.7 ± 0.5	<0.001
Symptom improvement (0–10), points	3.6 ± 1.2	1.1 ± 0.9	<0.001
Time-to-effective therapy, h (mean ± SD)	10.7 ± 6.2	22.9 ± 10.4	<0.001
Early IV-to-oral switch ≤72 h, n (%)	42 (55.3)	6 (12.0)	<0.001
Early catheter removal ≤48 h ^†^, n/N (%)	19/28 (68.0)	9/29 (31.0)	0.005

^†^ Among patients with CAUTI at admission. Abbreviations: CRP, C-reactive protein; IV, intravenous; ECR, early clinical response.

**Table 5:** Multivariable predictors of 72 h non-response (ECR−).

Variable	Adjusted OR (95% CI)	*p*-Value
CAUTI (yes vs. no)	1.9 (1.1–3.2)	0.02
Time-to-effective therapy (per 6 h)	1.5 (1.2–2.0)	<0.001
Baseline CRP (per 20 mg/L)	1.3 (1.0–1.7)	0.048
Diabetes (yes vs. no)	1.8 (1.0–3.3)	0.047
Early catheter removal ≤48 h	0.5 (0.3–0.9)	0.02
Early IV-to-oral switch ≤72 h	0.4 (0.2–0.8)	0.01

Model fit: Nagelkerke R^2^ = 0.41; Hosmer–Lemeshow *p* = 0.39. Abbreviations: OR, odds ratio; CI, confidence interval; CAUTI, catheter-associated UTI; CRP, C-reactive protein.

**Table 6:** Spearman correlation matrix for key continuous variables (n = 126).

Variable Pair	Spearman’s ρ	*p*-Value
CRP clearance (%) vs. length of stay (days)	−0.52	<0.001
Time-to-effective therapy (h) vs. length of stay (days)	0.41	<0.001
Baseline CRP (mg/L) vs. length of stay (days)	0.28	0.002
CRP clearance (%) vs. time-to-effective therapy (h)	−0.36	<0.001
Age (years) vs. length of stay (days)	0.18	0.048

Note: Negative ρ indicates inverse association (greater clearance, shorter stay).

**Table 7:** Time-to-effective therapy (TTE) strata vs. outcomes (n = 126).

TTE Stratum	N	ECR+ (%)	LOS, Mean (SD), Days
≤12 h	52	84.60%	10.2 (2.9)
13–24 h	42	61.90%	11.6 (3.5)
>24 h	32	25.00%	13.8 (4.1)

Global tests: LOS by strata (Kruskal–Wallis) H = 9.89, *p* = 0.007; ECR distribution by strata (χ^2^) χ^2^ = 34.24, df = 2, *p* < 0.001. Pairwise LOS (Mann–Whitney with Bonferroni): ≤12 h vs. >24 h *p* = 0.0069; ≤12 h vs. 13–24 h *p* = 0.322; 13–24 h vs. >24 h *p* = 0.147.

**Table 8:** Among CAUTI patients (n = 57): early catheter removal (≤48 h) vs. 72 h response.

Group	N	ECR+ (n, %)
Early removal ≤48 h	28	19 (67.9%)
No early removal	29	9 (31.0%)

Effect size and tests: Fisher’s exact *p* = 0.008; Risk Ratio (RR) = 2.19; 95% CI 1.20–3.98.

## Data Availability

Data availability is subject to hospital approval.
